# Relationship between long non-coding RNAs and Hippo signaling pathway in gastrointestinal cancers; molecular mechanisms and clinical significance

**DOI:** 10.1016/j.heliyon.2023.e23826

**Published:** 2023-12-22

**Authors:** Farimah Fayyaz, Zahra Shokati Eshkiki, Amir Reza Karamzadeh, Zahra Moradi, Faezeh Kaviani, Abolfazl Namazi, Roya Karimi, Seidamir Pasha Tabaeian, Fatemeh Mansouri, Abolfazl Akbari

**Affiliations:** a Colorectal Research Center, Iran University of Medical Sciences, Tehran, Iran; b Alimentary Tract Research Center, Clinical Sciences Research Institute, Imam Khomeini Hospital, Ahvaz Jundishapur University of Medical Sciences, Ahvaz, Iran; c Occupational Medicine Research Center, Iran University of Medical Sciences, Tehran, Iran; d Department of Genetic, Faculty of Sciences, Qom Branch, Islamic Azad University, Qom, Iran; e Young Researchers and Elite Club, Qom Branch, Islamic Azad University, Qom, Iran; f Department of Internal Medicine, School of Medicine, Iran University of Medical Sciences, Tehran, Iran

**Keywords:** Long non-coding RNA, Hippo, Signaling pathway, Gastrointestinal cancers, Diagnosis, Prognosis

## Abstract

Long non-coding RNAs (lncRNAs) play a significant biological role in the regulation of various cellular processes such as cell proliferation, differentiation, apoptosis and migration. In various malignancies, lncRNAs interplay with some main cancer-associated signaling pathways, including the Hippo signaling pathway to regulate the various cellular processes. It has been revealed that the cross-talking between lncRNAs and Hippo signaling pathway involves in gastrointestinal (GI) cancers development and progression. Considering the clinical significance of these lncRNAs, they have also been introduced as potential biomarkers in diagnostic, prognostic and therapeutic strategies in GI cancers. Herein, we review the mechanisms of lncRNA-mediated regulation of Hippo signaling pathway and focus on the corresponding molecular mechanisms and clinical significance of these non-coding RNAs in GI cancers.

## 1 Introduction

### 1.1 background

Gastrointestinal (GI) cancer is one of foremost cancer-related public health problem worldwide with greatly characterized by poor prognosis.**. GI cancers account for approximately 5 million new cases of cancers and 3 million deaths worldwide in 2020. The highest liver, esophageal, and gastric cancer rates are reported in Asia, while pancreatic and colorectal cancers were more prevalent in Europe in 2020** [1]**. The incidences of liver, pancreatic, and colorectal cancers are increasing in most countries** [2,3]**. Identifying novel, sensitive and specific cancer biomarkers for early diagnosis and estimating the prognosis of gastrointestinal cancers have developed particularly owned by more detailed discovery of cancer underlying molecular signaling pathways. Dysregulation of various signaling pathways and corresponding molecular mechanisms driven by numerous transformed and altered oncogenes and tumor suppressors have been described to be involved in GI development and progression** [4,5]**. Also, epigenetic alterations and dysregulation of DNA repair genes have been frequently involved in GI cancers. In addition to well-known signaling pathways, increasing evidences have demonstrated that non-coding RNAs, including long non-coding RNAs (lncRNAs) also play a fundamental regulatory role in the development and progression of GI cancers. LncRNAs have been confirmed to involve in GI cancers through interactions with numerous biological molecules. Moreover, these molecules may be served as potential molecular diagnostic and prognostic biomarkers as well as targets for GI cancers** [4,5] Table 1.

**Table 1:** LncRNAs that regulate gastrointestinal cancers by targeting Hippo signaling pathway; molecular mechanisms and clinical significance.

GI Cancer type	LncRNA	Regulatory molecular mechanism in Hippo signaling	Clinical significance	Reference
Intrahepatic Cholangiocarcinoma (ICC)	MNX1-AS1	Enhances the expression of MNX1 by recruiting transcription factors c-MYC and MAZ, suppressing the Hippo pathway and increasing ICC tumorigenesis and progression.		(13)
	PAICC	Promotes the proliferation and invasion of ICC through YAP1 regulation-mediated Hippo signaling pathway by sponging miR-141-3p/27A-3p.	Positively correlation with poor prognosis	(14)
Head and Neck Squamous Cell Carcinoma (HNSCC)	WWTR1-AS1		Positively correlation with poor outcomes in HNSCC patients	(17)
	LEF1-AS1	Interacts with LATS1 and inhibits its binding to MOB, which led to hindering YAP1 phosphorylation, inactivating Hippo signaling pathway in OSCC.	Positively correlation with poor outcomes in HNSCC patients in OSCC patients	(18)
	LINC01315	Competitively binds to miR-211, resulting in up-regulation of DLG3 expression. Increase proliferation, migration, and invasion and suppresses apoptosis in OSCC cells.		(19)
	LUADT1	Promotes cell proliferation, migration, and invasion by influencing miR-1207-5p.		(22)
Hepatocellular Carcinoma (HCC)	MALAT1	YAP protein induces MALAT1 transcription through the TCF/β-catenin element located in the MALAT1 promoter region, leading to tumor growth.		(25)
	uc.134	Activates Hippo signaling by inhibiting CUL4A translocation from the nucleus to the cytoplasm, increasing LATS1 stability and reducing YAP target genes expressions.	Associates with poor overall survival in HCC patients	(26, 27)
	PVT1			(28)
	UCA1	Induces apoptosis		(29)
	CRNDE	Interacts with SUZ12, EZH2, and SUV39H1 directly targets the tumor suppressor genes, LATS2 and CELF2. Is mediated by LATS2 and Hippo pathway so that LATS2 overexpression can inhibit the effect of CRNDE on the progression of HCC.	Associates with poor prognosis	(34)
	LOC107985656	Inhibits HCC cell proliferation and metastasis, its expression. Activates the Hippo pathway by regulating the expression of LATS1 through the miR-106b-5p/LATS1 axis	Negatively associates with poor prognosis	(36)
Pancreatic Cancer (PC)	GAS5	Inhibits MST1 protein and decreases YAP and TAZ phosphorylation, Hippo signaling inactivation, and promotes tumor growth *in vivo*		(40)
	UCA1	Induces YAP activation and binds to MOB1, LATS1, and YAP, forming a ribonucleoprotein complex.	Associates with poor prognosis	(42)
	MALAT1	Promotes proliferation, migration and invasion, and inhibits apoptosis *in vitro.*		(43)
	MVIH	Promotes microvascular invasion, proliferation and migration.	Associates with poor prognosis	(48)
	MUF (LINC00941)	Interacts with MST1, leading to the MST1 dephosphorylation by protein phosphatase 2A (PP2A) and increases YAP1 nuclear localization, promoting cell proliferation and metastasis.	Correlates with poor prognosis	(55)
Gastric Cancer (GC)	LincRNA P21	Inhibits cell growth, proliferation, migration, invasion and epithelial-mesenchymal transition (EMT). Decreases the YAP protein and mRNA level and YAP nuclear translocation.	Negatively associates with higher invasion depth grade and cancer metastasis	(58)
	AP000695.6 RP11-108M12.3 RP11-108M12.3		AP000695.6 and RP11-108M12.3 associates with better OS, and CYP4A22-AS1 with poor OS	(59)
	LINC00662	Promotes tumorigenesis by reducing YAP1 expression and inactivating Hippo pathway through targeting miR-497-5p.	Associates with poor prognosis	(60)
	HCG18	Enhances the proliferation, migration and invasion of GC cells through regulating miR-141-3p.	Associates with poor prognosis	(63)
	FER1L4	Activates the Hippo pathway through CXCR4/CXCL12 axis, suppressing the proliferation, invasion, migration, and metastasis of GC cells.	Associates with clinicopathological characteristics	(65)
	LINC00649	Inhibits apoptosis, enhances cell proliferation, migration, and EMT process *in vitro*, and promotes tumor growth *in vivo* through miR-16-5p downregulation and YAP1 upregulation.		(69)
	RP11-323N12.5	c-Myc acts as the link of RP11-323N12.5 affecting YAP1 gene promoter, promoting RP11-323N12.5 transcription in GC cells through TEAD1 binding site in RP11-323N12.5 promoter. Contributes to the upregulation of AXL, Survivin or MMP9 that enhance cell proliferation, migration and invasion via YAP1.	Associates with higher GC stages and worse disease-free survival	(70)
Colorectal Cancer (CRC)	B4GALT1-AS1	Promotes colon cancer stemness via binding to YAP and translocating it to nucleus to increase its transcriptional activity		(71)
	GAS5	Inhibits the YAP-mediated expression of YTHDF3, suppressing CRC cell proliferation, invasion, and decreases tumor growth.		(72)
	USP2-AS1	Upregulates the Hippo pathway target genes, CTGF, CYR61, and SOX9	Associates with tumor grade and stage	(73)
	LINC00152	Upregulates FSCN1 via sponging with miR-185-3p and miR-632, and promotes cell proliferation, invasion, and metastasis of CRC cells.	Associates with poor overall survival of patients	(74)
	SNGH11	Increases the EMT markers, including E-cadherin overexpression and N-cadherin downregulation. Promotes cell proliferation, migration and invasion.	As a diagnostic tool with a satisfactory accuracy in diagnosing CRC at early stages as well as advanced stages.	(75)
	AGAP2-AS1	Enhances CRC cell proliferation, migration and invasion.		(81)
	LINC-PINT	Enhances CRC cell apoptosis, and inhibits the cell proliferation, migration and invasion,		(81)
Gallbladder Cancer (GBC)	MNX1-AS1	Recruits ubiquitin specific peptidase 16 (USP16) and promote insulin-like growing factor 2 mRNA-binding protein 3 (IGF2BP3) activation. Promotes the proliferation and metastasis *in vitro* and *in vivo*.	Associates with poor survival of patients, as a valuable tool for diagnostic and therapeutic strategies of GBC	(82)

**Abbreviations:** CTGF; connective tissue growth factor, CYR61; cysteine-rich 61, GI; gastrointestinal, HNSCCs; head and neck squamous cell carcinomas, MST1/MST2; mammalian STE20-like protein kinase 1/2, LATS1/2; large tumor suppressor 1/2 (and LATS2), YAP; Yes-associated protein, TAZ; transcriptional co-activator with PDZ-binding motif, TEAD; transcriptional enhanced associate domain, ceRNA; competing endogenous RNA, ICC; intrahepatic cholangiocarcinoma, HCC; hepatocellular carcinoma, MNX1-AS1; motor neuron and pancreas homeobox 1-antisense RNA1, TCGA; The Cancer Genome Atlas, GEO; Gene Expression Omnibus, PAICC; prognostic-associated ICC, LEF1-AS1; lymphoid enhancer-binding factor 1 antisense RNA 1, OSCC; oral squamous cell carcinoma, LUADT1; lung adenocarcinoma-related transcript 1, NPC; nasopharyngeal carcinoma, MALAT1; metastasis-associated lung adenocarcinoma transcript 1, PVT1; plasmacytoma variant translocation 1, UCA1; urothelial cancer associated 1, CRNDE; colorectal neoplasia differentially expressed, PRC2; polycomb repressive complex 2, GAS5; growth arrest-specific 5, EMT; epithelial-mesenchymal transition, HCG18; HLA complex group 18, FER1L4; Fer-1-like family member 4, FSCN1; Fascin actin-bundling protein 1, SNGH11; small nucleolar RNA host gene 11, AGAP2-AS1; ankyrin repeat and PH domain 2 antisense 1, MNX1-AS1; motor neuron and pancreas homeobox1 antisense RNA 1.

The Hippo signaling pathway, as a well-recognized conserved signaling pathway, has been characterized to control tissue growth and organ size in physiological development, regeneration, and pathological conditions, including cancer. Initially, it was identified in Drosophila [6,7], but determining its conservation in mammals led to valuable discoveries of its role in cancer pathogenesis [8]. Accumulating evidences have emphasized the significance of Hippo signaling pathway in GI tissue homeostasis, while its aberration may be involved in GI cancer development and progression. A number of studies have revealed the biological role of Hippo pathway in GI cancer-related signaling pathways. In this regard, the Hippo signaling pathway effectors may interplay with common cancer signaling pathways to promote tumorigenesis [8]. Here, we review the biological role of this signaling pathway and its crosstalk with lncRNAs and highlight the molecular mechanisms and clinical significance of the crosstalking in the development of GI cancers.

### 1.2 Hippo signaling pathway; cell proliferation and tumorigenesis

The Hippo signaling pathway was first described as an imperative and conserved regulatory pathway in cell developmental processes, including organ size, tissue homeostasis, cell proliferation and apoptosis [6,7]. **The central axis of Hippo signaling pathway consists of various proteins, including mammalian STE20-like protein kinase 1 and 2 (MST1 and MST2) and large tumor suppressor 1 and 2 (LATS1 and LATS2) that promote Yes-associated protein (YAP) and transcriptional co-activator with PDZ-binding motif (TAZ) phosphorylation and accumulation in the cytoplasm** [6,7]**. YAP/TAZ may be affected by phosphorylation at different sites by upstream kinases, inducing the expression of target genes via binding to transcriptional enhanced associate domain (TEAD) protein family. In this manner, this signaling may involve in a variety of cellular processes, including cell growth, differentiation and proliferation. Therefore, YAP and TAZ phosphorylation inhibits their tumorigenic potential and regulation of TEAD and SMAD transcription factors** [9]**.**

**When Hippo pathway is activated by stimuli, MST1/2 kinase is phosphorylated and subsequently phosphorylates MST1/2. Then, phosphorylated MST1/2 kinase induces phosphorylation of salvador homolog 1 (SAV1) to form a heterotetramer to more increase the LATS1/2 phosphorylation** [6,7]**. Activated LATS1/2 causes inactivation or degradation of YAP/TAZ through ubiquitination, and thus inhibits the transcription of downstream genes. In other words, when the Hippo pathway is inactivated, translocation of YAP/TAZ to the nucleus and binding to enhancer elements result in transcription of target genes** [6,7]**. The interaction between** Hippo signaling pathway and vital target genes plays an imperative role in the regulation of various biological processes of vertebrate cells. **An increasing number of studies have highlighted a critical role of Hippo pathway in the regulation of cell proliferation, apoptosis, and metastasis** [6,7]**.**

### 1.3 long non-coding RNAs (lncRNAs): mechanisms and biological functions in cancer development and progression

**LncRNAs are defined as RNA molecules longer than 200 nucleotides that modulate gene expressions at different levels, including transcriptional and post-transcriptional levels** [10]**. They can directly regulate gene transcription and recruit transcription factors or interact with mRNAs and proteins** [11,12]**. Several lncRNAs function as competing endogenous RNAs (ceRNAs), whose regulation of mRNAs depends on microRNAs (miRNAs). In other words, lncRNAs can sponge and sequester miRNAs and prevent their regulation of mRNAs** [13]**.** LncRNAs have also been reported to interact with splicing factors and heterogeneous nuclear ribonucleoprotein (hnRNP) family proteins to regulate mRNA alternative splicing. **The action mode of epigenetic regulations by lncRNAs most commonly includes chromatin remodeling or histone modification** [[13], [14], [15]]**. Indeed, lncRNAs are involved in regulating histone modifications at the chromatin level. In this regard, lncRNA can function as a framework that complex with modification enzymes, thereby regulating histone modifications such as methylation and acetylation. LncRNAs also can regulate DNA methylation by interacting with DNA methyltransferase at the DNA level** [[13], [14], [15]]**. Other molecular mechanisms include interaction with the transcriptional effectors to regulate gene expression, direct participation in the post-transcriptional regulation of mRNAs, regulation of RNA editing, activation of Dicer complex and induction of non-coding RNA-based regulatory machinery. It has been well documented that lncRNAs play a pivotal functional role in key cellular processes including, cell proliferation, apoptosis and differentiation. A number of studies have revealed the dysregulation of expression and indicated the biological role of lncRNAs in various cancers initiation and progression** [13]**.**

Cancer-associated lncRNAs are categorized as oncogenic and tumor-suppressive lncRNAs based on their roles in tumor cell proliferation, invasion, epithelial-mesenchymal transition (EMT), cancer stem cell (CSC) maintenance and drug resistance [[13], [14], [15]]. From a clinical point of view, it has also been shown in several studies that the expression pattern of these molecules change in cancer patients compared to healthy individuals, which point to their clinical values. Because of biological and clinical significance of lncRNAs in cancers, they have been indicated as diagnostic and prognostic biomarkers and provided promising targets for therapeutic strategies [[13], [14], [15]].

### 1.4 The role of lncRNAs in gastrointestinal cancers

**Fig. 1:**
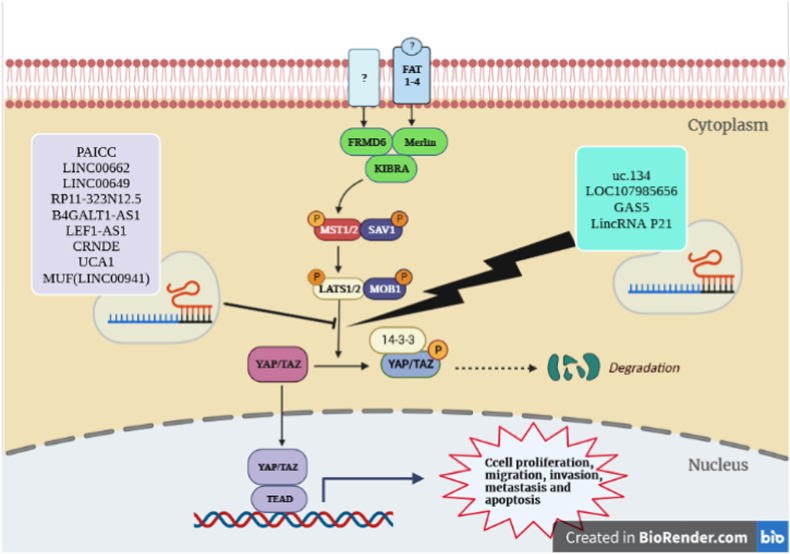
**Regulation of Hippo signaling pathway by lncRNAs in gastrointestinal cancers**. Activating Hippo-related receptors triggers the signaling pathway. Phosphorylated MOB recruits LATS (together with SAV and MST1/2), and LATS then is phosphorylated and activated. Then, activated LATS phosphorylates YAP/TAZ and promote their cytoplasmic localization by creating a binding site for 14-3-3 proteins. The heterodimer of YAP/TAZ and TEAD can transduce a signal to nucleus for regulation of gene transcription. LncRNAs commonly **bind or regulate LATS and YAP1/TAZ, translocating phosphorylated form into nucleus ultimately results in** regulation of cellular processes such as proliferation, migration and apoptosis.

**Fig. 2:**
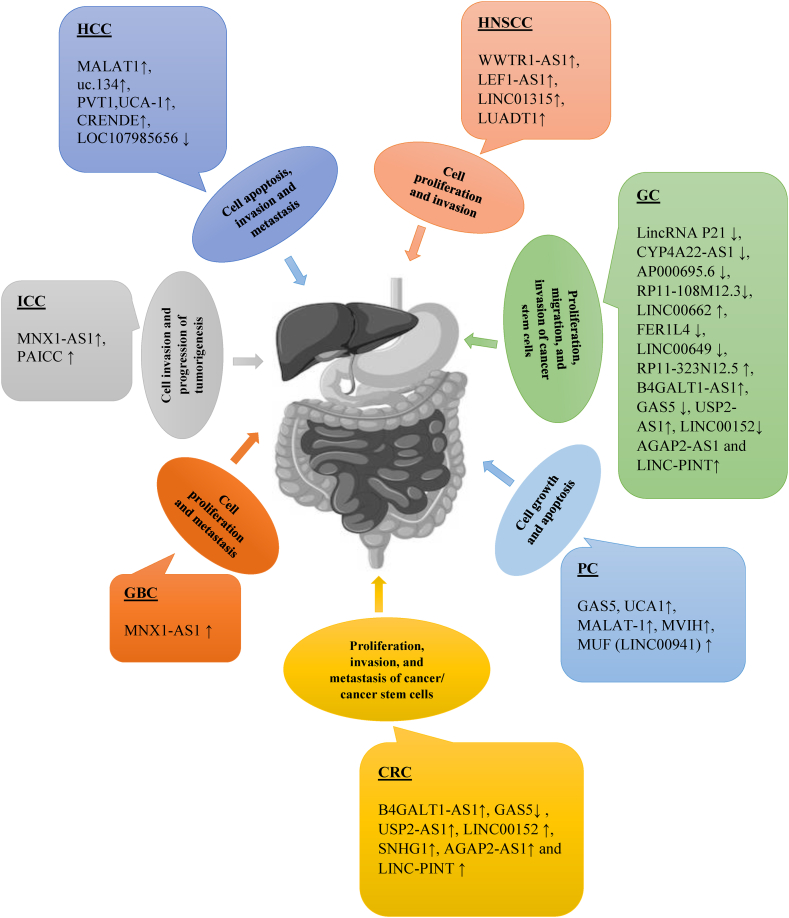
Relationship of lncRNAs and Hippo pathway in gastrointestinal cancers development and progression. Various lncRNAs may dysregulate and contribute to GI cancers development and progression by regulating Hippo signaling pathway. Cross-talking and regulatory function of lncRNAs may control cell proliferation, migration, apoptosis and metastasis of GI cancers. Abbreviations, CRC; colorectal cancer, GBC; gallbladder cancer, HNSCCs; head and neck squamous cell carcinomas, ICC; intrahepatic cholangiocarcinoma, HCC; hepatocellular carcinoma, MNX1-AS1; motor neuron and pancreas homeobox 1-antisense RNA1, LEF1-AS1; lymphoid enhancer-binding factor 1 antisense RNA 1, LUADT1; lung adenocarcinoma-related transcript 1, GC; gastric cancer, MALAT1; metastasis-associated lung adenocarcinoma transcript 1, PVT1; plasmacytoma variant translocation 1, UCA1; urothelial cancer associated 1, CRNDE; colorectal neoplasia differentially expressed, PRC2; polycomb repressive complex 2, GAS5; growth arrest-specific 5, HCG18; HLA complex group 18, FER1L4; Fer-1-like family member 4, FSCN1; Fascin actin-bundling protein 1, SNGH11; small nucleolar RNA host gene 11, AGAP2-AS1; ankyrin repeat and PH domain 2 antisense 1, MNX1-AS1; motor neuron and pancreas homeobox1 antisense RNA1, PC; pancreatic cancer.

**The prominent role of lncRNAs in gene expression highlights their potential to act as suppressors or promoters of proliferation, invasion, and metastasis of GI tumors.** The cell proliferation, apoptosis, invasion, cell cycle, EMT, CSC and drug resistance may be regulated by lncRNAs in GI tract. The growing evidences from experimental, functional and computational studies indicate that lncRNAs play substantial roles in GI tumorigenesis. Furthermore, aberrant expression of lncRNAs has been reported to be correlated with the clinicopathological parameters, indicating them as a class of auspicious biomarkers in GI cancers [13,14]. **As a modulator of various cellular processes involved in GI tumorigenesis, the Hippo pathway could be a target of lncRNAs (**Fig. 1**). The present study reviews the cross-talk and interaction mechanisms between lncRNAs and the Hippo signaling pathway and highlighted clinical significance in GI cancers (**Fig. 2**).**

## 2 cross-talk between lncRNAs and the Hippo signaling pathway in gastrointestinal cancers

Functional and mechanistic studies have been confirmed that several lncRNAs maigh regulate Hippo signaling pathway through targeting some molecular effectors. Hippo pathway cascades also reciprocally might regulate the lncRNAs synthesis machinery. The molecular crosstalking between lncRNAs and Hippo signaling pathway has been established in various GI cancers. This regulatory mechanism eventually influences GI cancer development and progression, demonstrating a multifaceted biological connection between lncRNAs and Hippo signaling pathway.

### 2.1 head and neck squamous cell carcinoma

**Head and neck squamous cell carcinoma (HNSCC) represents the seventh most common cancer worldwide, with approximately 930,000 new cases in 2020** [16]**. The history of smoking, alcohol drinking, and human papillomavirus infection are proven risk factors for HNSCC** [[17], [18], [19]]**. Because of variety of clinical symptoms at the early stages and the lack of an applicable diagnostic biomarker,** identification of molecular mechanisms in order to find diagnostic and therapeutic biomarkers is needed**.** The vast majority of malignancies, which are almost exclusively squamous cell carcinomas (SCCs), originate from of the head and neck that are part of the upper aero digestive tract (UADT). UADT is structured such that it consists of many primary sites, each of which is further split into multiple anatomic sub sites. Some examples of these sub sites are the oropharynx and the nasopharynx which are considered as parts of gastrointestinal tract. **The Hippo signaling pathway has been confirmed to involve in HNSCC development and progression. In this regard, the Hippo regulatory role of lncRNAs has been reported in HNSCC tumorigenesis.**

#### 2.1.1 LncRNA WWTR1-AS1

**LncRNA WW domain-containing transcription regulator-1 antisense RNA 1 (WWTR1-AS1) is a natural antisense transcript (NAT). WWTR1-AS1 is significantly overexpressed in 37 HNSCC tissues compared to adjacent normal tissue, and it could promote cell proliferation and invasion in** HNSCC cell lines SCC4, SCC9, SCC25, FaDu, and Cal27**. It has also been revealed that the overexpression of both lncRNA WWTR1 and WWTR1 mRNA, as its correspondence with TAZ, are associated with poor outcomes in 37 HNSCC patients. Therefore, lncRNA WWTR1-AS1 possibly corresponds to WWTR1. Moreover, the overexpression of this lncRNA was significantly associated with WWTR-1 expression in the TCGA-HNSCC dataset and clinical samples. Based on combination analyses, WWTR-1 was introduced as a recognized downstream regulatory target in the Hippo pathway** [20]**.**

#### 2.1.2 LncRNA LEF1-AS1

**The expression levels and functional role of lymphoid enhancer-binding factor 1 antisense RNA 1 (LEF1-AS1) have been investigated in oral squamous cell carcinoma (OSCC). The findings showed that LEF1-AS1 was significantly overexpressed in 20 OSCC tissues compared to the adjacent non-tumor tissues. LEF1-AS1 overexpression was associated with poor outcomes in OSCC patients. In this regard, knockdown of LEF1-AS1 inhibited cell survival and proliferation, promoted apoptosis, and suppressed tumor growth of** CNE-1, CNE-1/T, HNE-2, and HNE-2/T cells**. Furthermore, LEF1-AS1 influenced the Hippo signaling pathway by interacting with LATS1 and inhibiting its binding to MOB, which led to hindering YAP1 phosphorylation. Therefore, it was indicated that LEF1-AS1 inactivates Hippo signaling pathway in OSCC** [21]**.**

#### 2.1.3 LINC01315

**The expression of long intergenic non-protein coding RNA 1315 (LINC01315) is down-regulated in OSCC tissues** [22]**. Based on bioinformatic analysis of** the GSE45238 dataset of the GEO database, it was hypothesized that miR-211 might interact with LINC01315 and affect OSCC development and progression. The miR-211 contains complementary sequences for LINC01315 and disks large homolog 3 (DLG3). The evaluation of OSCC and adjacent tissues demonstrated that the expression levels of DLG3 and LINC01315 are significantly higher, and the expression level of miR-211 is significan**tly lower in the OSCC tissues** [23]**. Further analysis showed tha**t LINC01315 competitively binds to miR-211, resulting in up-regulation of DLG3 expression. Also, LINC01315 knockdown enhanced proliferation, migration, and invasion and supp**ressed apoptosis in OSCC** cell lines SAS, SCC25, HN4, HN6, CAL-27, and NHOK**. A previous study pointed to a possible relationship between up-regulation of DLG3 and the Hippo pathway in breast cancer** [23]**. The results showed that down-regulated DLG3 was associated with down-regulation of MST1, MST2, and LATS 1, and up-regulation and nuclear translocation of YAP protein** [24]**.**

#### 2.1.4 LncRNA LUADT1

**One lncRNA named lung adenocarcinoma-related transcript 1 (lncRNA LUADT1) has been revealed to involve in nasopharyngeal carcinoma (NPC) progression. Clinical investigations discovered that lncRNA LUADT1 and TEAD1 were significantly overexpressed, and miR-1207-5p was significantly down-regulated in 79 NPC tissues and 4 cell lines** HONE-1, HNE-1, CNE1, CNE2. **The bioinformatic and luciferase reporter assay showed lncRNA LUADT1 and TEAD1 have binding sites for miR-1207-5p. The knockdown of lncRNA LUADT1 inhibited cell proliferation, migration, and invasion, which was reversed by the inhibitory effect of miR-1207-5p. The effect of lncRNA LUADT1 in NPC progression was also verified *in vivo*. Regarding the Hippo pathway, LncRNA LUADT1 knockdown results in the LATS1 protein up-regulation and YAP1 and TAZ down-regulation. However, miR 1207-5p down-regulation has a contrary effect** [25]**.**

### 2.2 hepatocellular carcinoma

**HCC, the most common primary liver cancer, is the third leading cause of cancer death** [16]**. In the recent decade, the early treatment of HCC has progressed dramatically, improving the prognosis. Since most patients are diagnosed in later stages of the disease and are not eligible for surgical resection and conventional treatments** [26]**, identifying underlying molecular processes in HCC progression could develop novel diagnostic and therapeutic strategies. The Hippo signaling pathway has been confirmed to involve in HCC development and progression through regulating key Hippo-related genes, including *Sav1, Mst1/2, Mob1a/b* and *Lats1/2.* Moreover, the Hippo regulation role of lncRNAs has been reported in HCC tumorigenesis mainly by affecting YAP/TAZ** [27]**.**

#### 2.2.1 LncRNA MALAT1

**Metastasis-associated lung adenocarcinoma transcript 1 (MALAT1) is a lncRNA with high expression in many tumors and is associated with an increased risk of metastasis** [27]**. A recent study explored the relationship between YAP protein, serine/arginine-rich splicing factor 1 (SRSF1), and MALAT1 in 112 HCC patients and cell line** HepG2**. It was demonstrated that YAP protein induces MALAT1 transcription through the TCF/β-catenin element located in the MALAT1 promoter region, which leads to tumor growth. Furthermore, SRSF1 down-regulates MALAT1 expression by facilitating the translocation of intra-nucleus accumulated YAP to the cytoplasm and accelerating MALAT1 degradation** [28]**.**

#### 2.2.2 LncRNA uc.134

**The lncRNA uc.134 was found with a low expression in HCC cell line with a high metastatic potential named HCCLM3. The expression of uc.134 was shown to be significantly decreased in** 170 **paraffin-embedded HCC compared to the adjacent tissues, and the lower uc.134 expressions was significantly associated with poor overall survival in HCC patients. Moreover, the reduction of mRNA levels of YAP target genes was observed in the overexpression of uc.134. The relationship between uc.134, LATS1, and pYAP was confirmed in 90 paraffin-embedded HCC samples. The investigation of the molecular mechanisms of uc.134 in HCC progression verified that CUL4A protein, which is an E3 ligase targeting LATS1 protein for degradation and ubiquitination** [29]**, bound to uc.134 to form an RNP complex. The overexpression of uc.134 was associated with CUL4A accumulation in the nucleus, while in uc.134 knockdown, CUL4A was observed in the cytoplasm. Thus, it was confirmed the interaction of LATS1 and CUL4A proteins in HCC cells may result in more stability of LATS1 protein in cells transfected with CUL4A and uc.134 compared to CUL4A alone. Altogether, the findings suggested that lncRNA uc.134 activates Hippo signaling by inhibiting CUL4A translocation from the nucleus to the cytoplasm, increasing LATS1 stability and reducing YAP target genes expression** [30]**.**

#### 2.2.3 LncRNA PVT1

**LncRNA plasmacytoma variant translocation 1 (PVT1) is associated with the development and progression of cancers.** Functional studies on HCC cell line SMMC-7721 demonstrated that lncRNA PVT1 may promote liver cancer progression by inhibiting histone methylation on the MYC promoter, signifying it as potential target for therapeutic strategies of the malignancy. **The potential genes associated with PVT1 in HCC have been investigated by bioinformatic analysis** [[13], [14], [15]]**.** Moreover, PVT1 has been revealed to upregulate in HCC and significantly correlate with clinicopathological features of the patients, indicating it as a valuable diagnostic biomarker. Further analysis showed that PVT1 may function as an oncogene in HCC development probably via regulating the Hippo pathway. Altogether, **findings indicated that DLC1 (deleted in liver cancer 1) and PVT1 genes are potentially involved in the Hippo signaling pathway** [31]**.**

#### 2.2.4 LncRNA UCA1

**Urothelial cancer associated 1 (UCA1) is one lncRNA upregulated in various cancers. The biological role of lncRNA UCA1 has been explored in HCC. After confirming significant UCA1 upregulation in 41 HCC tissues in comparison with adjacent tissues and a positive correlation of UCA1 overexpression with advanced stages of HCC, UCA1 was knocked down in HCC cell lines** Huh-7, Hep3B, HepG2 and SMMC7721, **resulting in inhibition of cell growth and induction of apoptosis. In this regard, the KEGG pathway analysis also demonstrated that UCA-related genes were significantly enriched in the Hippo signaling pathway** [32]**.**

#### 2.2.5 LncRNA CRNDE

**Colorectal neoplasia differentially expressed (CRNDE) have some oncogenic properties in different cancers** [[33], [34], [35], [36]]**. In several investigations, it has been demonstrated that CRNDE was significantly upregulated in 47 HCC tissues, and its overexpression was associated with poor prognosis** [37]**. The knockdown of CRNDE inhibited cell proliferation, migration, and chemoresistance in** HCC cell lines C-9810, Bel-100, Bel-7405, Bel-7402, Huh-7, WRL68, SMMC-7721, HepG2**, and inhibited tumor growth and tumor metastasis *in vivo*. Moreover, to explore the CRNDE mechanism in HCC pathogenesis, the direct binding between CRNDE and SUZ12, EZH2, and SUV39H1 was documented. SUZ12 and EZH2 are subunits of polycomb repressive complex 2 (PRC2), which exert tumorigenic roles owing to histone methyltransferase activity and modulating target gene expression** [38]**. It has been validated that the complex of CRNDE with SUZ12, EZH2, and SUV39H1 directly targets the tumor suppressor genes, LATS2 and CELF2. Also, the regulation of CRNDE in HCC is mediated by LATS2 and Hippo pathway so that LATS2 overexpression can inhibit the effect of CRNDE on the progression of HCC** [37]**.**

#### 2.2.6 LncRNA LOC107985656

**The LOC107985656 is a recently reported lncRNA that its roles have been investigated in 30 HCC patients. The researchers demonstrated that LOC107985656 inhibits proliferation of HCC cell lines** Huh 7, SMMC-7721, HepG2.2.15 and HepG2. **Also, its expression was significantly downregulated in HCC tissues, and its low expression was associated with a poor prognosis. Moreover, LOC107985656 was shown to regulate the Hippo pathway. Although LOC107985656 knockdown did not affect YAP and TAZ mRNA levels in HCC cells, it significantly increased the YAP and TAZ protein expression and decreased the mRNA level of LATS1 and the levels of phosphorylated YAP and TAZ, leading to the inactivation of Hippo pathway. To investigate the underlying mechanism of LOC107985656 in regulating LATS1, the researchers predicted the miRNAs that may bind to LOC107985656 and LATS1 through the ceRNA mechanism. Ultimately, they showed that LOC107985656 activates the Hippo pathway by regulating the expression of LATS1 through the miR-106b-5p/LATS1 axis** [39]**.**

### 2.3 intrahepatic cholangiocarcinoma

**Intrahepatic cholangiocarcinoma (ICC) is the second most common primary liver malignancy after hepatocellular carcinoma (HCC). The incident rates of ICC are increasing annually** [40]**, followed by an increase in mortality rates** [41,42]**. Surgical resection is the only curative treatment option; however, the five-year overall survival rates are around 11–40 %** [43]**. Regarding systemic therapies, the standard of care includes gemcitabine and cisplatin as first-line, FOLFOX as second-line, and capecitabine as adjuvant therapy** [44]**. The data on target therapy in ICC is still limited, and the role of lncRNAs in this area needs to be explored.**

**A number of molecular mechanisms have been described for ICC tumorigenesis and metastasis. The Hippo signaling pathway has been confirmed to contribute into ICC development and progression. The regulatory role of lncRNAs has also confirmed to involve in the Hippo-mediated tumorigenesis process.**

#### 2.3.1 LncRNA MNX1-AS1

**In a recent study, the role of lncRNA MNX1-AS1, also known as motor neuron and pancreas homeobox 1-antisense RNA1, was investigated and MNX1-AS1/c-MYC and MAZ/MNX1/Ajuba/Hippo pathway was introduced. Initially, the analysis of RNA-seq data of The Cancer Genome Atlas (TCGA) and Gene Expression Omnibus (GEO) databases demonstrated that the expression of MNX1-AS1 and MNX1 were significantly upregulated and positively correlated in 33 ICC tissues. The *in vivo* and *in vitro* investigations showed that MNX1-AS1 might enhance the expression of MNX1 in ICC cell lines (**RBE, QBC939, and FRH0201) **by recruiting transcription factors c-MYC and MAZ. After that, MNX1 promotes Ajuba protein expression, suppressing the Hippo pathway. The inhibition of the Hippo pathway and YAP1 increase leads to ICC tumorigenesis and progression** [45]**.**

#### 2.3.2 LncRNA PAICC

**In another study, a competitive endogenous RNA (ceRNA) network of ICC was established. The analysis of the involved genes in the Hippo pathway in a GEO database showed that the expression of lncRNA-RP11**–**375I20.6, also named prognostic-associated ICC (PAICC) lncRNA, was upregulated in ICC. The expression analysis of PAICC and YAP1 showed that the expression levels of these genes were significantly higher in the 76 ICC tissues compared to adjacent healthy tissues** [46]**. Moreover, there was a significant positive correlation between PAICC and YAP1 expressions. In this regard, it was demonstrated that PAICC was significantly associated with poor prognosis in ICC patients, which gives insight into this lncRNA's oncogenic feature. The bioinformatic analysis showed that lncRNA-PAICC and PAICC have binding sites for miR-141-3p and miR-27a-3p thereby compete with these two miRNAs and down-regulates their expression in ICC cells. The dual-luciferase reporter assays confirmed that YAP1 is the downstream target gene of this ceRNA axis (PAICC/miR-141-3p and miR-27a-3p/YAP1). Thus, lncRNA-PAICC has an essential role in the proliferation and invasion of ICC through YAP1 regulation-mediated Hippo signaling pathway by sponging miR-141-3p/27A-3p** [46]**.**

### 2.4 gallbladder cancer

**Gallbladder cancer (GBC) is considered as a common and invasive of biliary tract malignancy with a poor prognosis diagnosis in late stages. Usually, traditional drug-based therapies are inefficient and the only curative approach for early-stage of the malignancy is whole surgical resection. Development of therapeutic strategies is remarkably reliant on the awareness from underlying molecular mechanisms and the early diagnostic biomarkers. Some epigenetic modulators such as miRNAs and lncRNAs have been revealed to involve in tumorigenesis and metastasis of GBC through regulating Hippo pathway, which could be valuable for diagnosis and therapy of the cancer** [47]**.**

#### 2.4.1 MNX1-AS1

A recent study examined the clinical and biological importance of lncRNA motor neuron and pancreas homeobox1 antisense RNA 1 (MNX1-AS1) in gallbladder cancer (GBC). The results indicated that the expression levels of lncRNA MNX1-AS1 were increased in the 33 tumor tissues. This overexpression of the lncRNA was revealed to be correlated with poor survival of GBC patients [48]. Moreover, it was demonstrated that MNX1-AS1 could promote the proliferation and metastasis of GBC cells (RBE and QBC939) *in vitro* and *in vivo*. The functional studies confirmed that TEA domain family member 4 (TEAD4) may promote the expression of MNX1-AS1. In this way, MNX1-AS1 may recruit ubiquitin specific peptidase 16 (USP16) and promote insulin-like growing factor 2 mRNA-binding protein 3 (IGF2BP3) activation. Based on the findings, this signaling pathway axis might promote the tumor development and progression. In this regard, lncRNA MNX1-AS1 has been indicated as a valuable tool for diagnostic and therapeutic strategies of GBC [48].

### 2.5 pancreatic cancer

**Pancreatic cancer (PC) is an aggressive malignancy, accounting for 466,000 global deaths in 2020, and it is projected to become the second leading cause of cancer death by 2030 in the United States** [49]**. The mortality/incidence ratio of as high as 94 % and the 5-year survival rate of 9 % give insight into pancreatic cancer's highly fatal nature. The stage at the time of diagnosis has a very significant effect on the survival rate of pancreatic cancer** [50]**. A timely diagnosis and treatment of pancreatic cancer patients could become possible by identifying novel molecular biomarkers. Several studies documented the Hippo signaling pathway in PC tumorigenesis. Also, it has been found the Hippo regulatory role of lncRNAs in PC development and progression.**

#### 2.5.1 LncRNA GAS5

**LncRNA growth arrest-specific 5 (GAS5) is a tumor-suppressive lncRNA that has an essential role in the tumorigenesis of various cancers** [51]**. Gao et al. predicted that miR-181c-5p could be a direct target of GAS5** [52]**. The miR-181c-5p was previously reported to be upregulated in pancreatic cancer, and it inhibited apoptosis and promoted chemoresistance by inactivating the Hippo signaling pathway** [53]**. Also, they demonstrated that GAS5 was downregulated, and miR-181c-5p was upregulated in drug-resistant pancreatic cancer cells** SW1990/GEM and PATU8988/5-FU **compared to drug-sensitive cells** SW1990 and PATU8988**. It was verified that GAS5 directly binds to miR-181c-5p and negatively regulates it. GAS5 upregulation led to MST1 protein overexpression and increased YAP and TAZ phosphorylation, Hippo signaling activation, and inhibited tumor growth *in vivo*. At the same time, miR-181c-5p upregulation compromised GAS5-induced MST1, p-YAP, and *p*-TAZ overexpression** [52]**.**

#### 2.5.2 LncRNA UCA1

**The biological role of lncRNA UCA1 has also been investigated in PC. It has been established that UCA1 was upregulated in pancreatic cancer tissue, and its overexpression was associated with enhanced migration and invasion of cancer cells and poor prognosis. The investigation of UCA1 functional significance in PC cell lines** BxPC-3, SW1990, PaTu8988, and PANC-1 **showed that UCA1 induces YAP activation and binds to MOB1, LATS1, and YAP, forming a ribonucleoprotein complex. It was demonstrated that UCA1 inhibits YAP phosphorylation and promotes YAP translocation to the nucleus. Also, YAP increased UCA1 expression in pancreatic cells, indicating a positive feedback between Hippo signaling and lncRNA UCA1** [54]**.**

#### 2.5.3 LncRNA MALAT1

**Zou et al. investigated the MALAT1 expression and its function in pancreatic cancer. They showed that lncRNA MALAT1 was upregulated in 15 pancreatic cancer tissues compared to the adjacent tissues. Furthermore, MALAT1 was demonstrated to promote proliferation, migration and invasion, and inhibit apoptosis *in vitro* (cell lines** AsPC-1, PANC-1 and BxPC-3)**, and promote tumor growth *in vivo*. The immunohistochemistry analysis showed that the expression levels of LATS1 and YAP1 were significantly higher in pancreatic cancer than adjacent tissue. The analyses of LATS1 and YAP1 and MALAT1 expression levels showed a negative correlation between LATS1 and MALAT1, and a positive correlation between YAP1 and MALAT1** [55]**.**

#### 2.5.4 LncRNA MVIH

**LncRNA associated with microvascular invasion in HCC (MVIH) is associated with microvascular invasion, proliferation and migration in various solid tumors** [[56], [57], [58], [59]]**. In a recent study, the molecular mechanism of MVIH was investigated in PC. The expression of lncRNA MVIH in 70 patients with pancreatic cancer showed that lncRNA MVIH expression was significantly higher in the tumor tissues compared to the adjacent tissues and its overexpression was associated with poor prognosis. *In vitro* evaluation demonstrated that lncRNA MVIH was overexpressed in pancreatic cancer cell lines (**PSN-1, AsPC-1, PANC-1 and BxPC-3) **and it promoted cell proliferation and invasion, and inhibited apoptosis. Moreover, the molecular mechanism of lncRNA MVIH and the involved signaling pathways were investigated. The RNA sequencing and bioinformatic analysis showed 196 differentially expressing genes (DEGs) in pancreatic cancer cell lines with overexpressed MVIH. Furthermore, KEGG enrichment analysis demonstrated that these DEGs were enriched in various cancer signaling pathways, including Hippo signaling pathway. The accordant DEGs associated with Hippo signaling pathways were SMAD4, PRKCZ, SNI2, FZD4, TGFB2, BIRC5, and AREG** [60]**.**

#### 2.5.5 LncRNA MUF (LINC00941)

**The MSC-upregulated factor (lncRNA MUF) is a new lncRNA that has been previously revealed to involve in various cancers and promote cell proliferation and metastasis** [[61], [62], [63], [64], [65], [66]]**. In pancreatic adenocarcinoma, the expression of lncRNA MUF is significantly higher in the tumor tissue compared to the adjacent normal tissue and its high expression correlates with poor prognosis** [67,68]**. One of the mechanisms of function of lncRNA MUF for promotion of pancreatic adenocarcinoma growth in cells** (PANC-1, SW1990, CFPAC-1, and BxPC-3**) is increasing aerobic glycolysis. The TEAD1 has been identified as the sole transcription factor of regulating glycolysis increased by lncRNA MUF transfection. Downstream genes of Hippo pathway were analyzed and demonstrated to be affected by this lncRNA. Furthermore, it was shown that lncRNA MUF interacts with MST1, leading to the MST1 dephosphorylation by protein phosphatase 2A (PP2A) and increasing YAP1 nuclear localization in pancreatic adenocarcinoma** [67]**.**

### 2.6 gastric cancer

Gastric cancer (GC) is a lethal malignancy of gastrointestinal tract with a high recurrence rate. The prevalence and mortality of GC are growing in undeveloped and developing countries. A number of attempts have been completed for identification of molecular mechanisms and supportive biomarkers for diagnosis, prognosis and therapy of GC [47]. **The regulatory feedback of lncRNAs/Hippo has also been revealed to involve in the GC tumorigenesis with a clinical significance** [69,70]**.**

#### 2.6.1 LincRNA P21

**The lincRNA P21 has been recognized as the direct transcriptional target of p53 that its low expression is linked with poor prognosis of several cancers** [71]**. In GC, lincRNA P21 was significantly downregulated in 40 tumor tissues in comparison with the adjacent normal tissue and its low expression was significantly associated with higher invasion depth grade and cancer metastasis. The *in vitro* study on biological function of lincRNA P21 showed that lincRNA P21 could inhibit cell growth, proliferation, migration, invasion and epithelial-mesenchymal transition (EMT) in GC cell lines** MGC-803 and MKN-45. **Further investigation on possible mechanism of lincRNA P21 in suppressing GC indicated that silencing lincRNA P21 in GC cell lines resulted in the YAP protein and mRNA level increase and YAP nuclear translocation** [72]**.**

#### 2.6.2 LncRNAs CYP4A22-AS1, AP000695.6, and RP11-108M12.3

**In 2018, it was published an article on a three lncRNA signature that evaluates the prognosis of patients with GC. The expression levels of the three lncRNAs in GC and adjacent normal tissue on TCGA database showed that** AP000695.6 a**nd RP11**–**108M12.3 were associated with better OS, and CYP4A22-AS1 with poor OS. A prognostic nomogram using the three lncRNA signature, age, TNM, stage, residual tumor, and risk score was established which showed a superior performance compared to the conventional TNM staging. Moreover, a predictive model by machine learning predicted the survival with satisfactory results. Finally, the KEGG pathway analysis demonstrated that the Hippo pathway is strongly linked to the three lncRNA risk score** [73]**.**

#### 2.6.3 LINC00662

**Recently, a novel GC-associated lncRNA**, named LINC00662, was identified using the TCGA database. The LINC00662 expression was significantly higher in GC tissues and cell lines. The higher expression of LINC00662 was also associated with poor prognosis of GC patients. The exploration of LINC00662 biological function showed that its knockdown in GC cell lines (MKN-45, MGC-803, SGC-7901, HGC-27, BGC-823, and AGS**) was associated with decreased cell proliferation and colony formation, and over sensitization of GC cells to 5-fluorouracil** [74]**. Regarding the Hippo pathway, th**e LINC00662 knockdown was correlated to the downregulation of CTGF, CYR61, and YAP1 mRNA and protein expression. Via bioinformatic analysis, five miRNAs were selected that potentially had binding sites for LINC00662. Among the selected miRNAs, the expression of miR-497-5p was significantly lower in 30 GC tissues, and after the LINC00662 knockdown, miR-497-5p was upregulated. Upon the transfection of GC cells with mi**R-497-5p, the YAP1 protein expression was significantly downregulated. Moreover, the luciferase reporter assay confirmed the direct binding of miR-497-5p to** LINC00662**. Altogether, it was concluded that** LINC00662 **may be a potential GC biomarker that promotes tumorigenesis by reducing YAP1 expression and inactivating Hippo pathway through targeting miR-497-5p** [75]**.**

#### 2.6.4 LncRNA HCG18

**Recent studies have demonstrated that lncRNA HLA complex group 18 (HCG18) might act as ceRNAs of numerous miRNAs and promote cancer progression through several signaling pathways. The lncRNA HCG18 expression was significantly higher in 79 GC tumors than in normal adjacent tissues with a correlation to poor prognosis** [[76], [77], [78]]**. Also, it has been shown that HCG18 knockdown in GC cell lines** AGS and MKN-28 **decreased tumor proliferation, migration, and invasion, and suppressed tumor growth and metastasis. A binding site between HCG18 and miR-141-3p was determined via bioinformatic analysis, which was validated by luciferase reporter assay. There was a correlation between HCG18 and miR-141-3p expressions in GC cells, in such a way that HCG18 overexpression promoted the expression of miR-141-3p. Furthermore, HCG18 enhanced the proliferation, migration and invasion of GC cells through regulating miR-141-3p. Indeed, the downregulation of HCG18 resulted in miR-141-3p inhibition and significantly decreased the WIPF1, YAP and TAZ mRNA and protein expression levels** [78]**.**

#### 2.6.5 LncRNA FER1L4

**LncRNA Fer-1-like family member 4 (FER1L4) was found to be downregulated in GC tissues and its expression is associated with clinicopathological characteristics** [79]**. To identify the underlying mechanisms of FER1L4 in GC,** the effect of FER1L4 overexpression on GC cell line SGC-7901 has been explored**. Findings demonstrated that FER1L4 overexpression suppressed proliferation, invasion, migration, and metastasis of GC cells. Moreover, the FER1L4 overexpression in GC cell lines led to the activation of Hippo pathway through CXCR4/CXCL12 axis. The role of FER1L4 in the GC cell proliferation and invasion through interacting with YAP and modulating Hippo pathway has been revealed. The results demonstrated that YAP reversed the inhibitory effect of FER1L4 overexpression on cell cycle, invasion, migration and lymphatic metastasis** [80]**.**

#### 2.6.6 LINC00649

**Long intergenic nonprotein coding RNA 649 has been reported as an lncRNA that functions in cancer initiation and progression. A key ceRNA signaling axis identified in GC was LINC00649/miR-16-5p** [[81], [82], [83]]**. (LINC00649) is an lncRNA that was p**reviously recognized as a prognostic biomarker of acute myeloid leukemia, prostate, and colorectal cancers. GC and adjacent normal tissues of 54 patients were evaluated by RT-qPCR and the results demonstrated that the expression of LINC00649 and YAP1 mRNA were significantly increased, while miR-16-5p expression was decreased in GC tissues. The functional experiments showed that LINC00649 overexpression in GC cell lines (MGC-803 and SGC-7901) resulted in inhibition of apoptosis, enhanced cell proliferation, migration, and EMT process in vitro, and promotion of tumor growth in vivo. They predicted that LINC00649 and YAP1 mRNA contain binding sites for miR-16-5p by bioinformatic analysis, which was conf**irmed by luciferase reporter assay. The knockdown of LINC0649 inhibited YAP1 mRNA and protein expression, which was reversed by silencing miR-16-5p. In fact,** LINC00649 **might promote GC through miR-16-5p downregulation and YAP1 upregulation** [69]**.**

#### 2.6.7 LncRNA RP11-323N12.5

**Another lncRNA that based on the TCGA database is the most upregulated lncRNA in GC, is RP11**–**323N12.5. Also, it was demonstrated that the high RP11**–**323N12.5 expression was significantly associated with advanced stages of GC and worse disease-free survival. The researchers have reported a correlation between the expression level of RP11**–**323N12.5 and Hippo-associated genes. Altogether, the biological regulatory role of lncRNA RP11**–**323N12.5 in Hippo signaling pathway was indicated** [74]**. The RP11**–**323N12.5 upregulation was confirmed in 67 paired human GC and normal adjacent tissues and it was correlated with the overexpression of either YAP1 mRNA or protein levels in GC tissues. The overexpression and knockdown experiments showed that RP11**–**323N12.5 may regulate YAP1 transcription. The transcription factor that had binding sites for both RP11**–**323N12.5 and YAP1 gene promoter was c-Myc, as a mediator for inducing YAP1 promoter** [74]**. Moreover, YAP1 activation could promote RP11**–**323N12.5 transcription in GC cell lines** MKN45 and MGC-803 **through TEAD1 binding site in RP11**–**323N12.5 promoter region. It has been revealed that lncRNA RP11**–**323N12.5 could contribute to the upregulation of AXL, survivin or MMP9 that enhance cell proliferation, migration and invasion via YAP1. Another influence of RP11**–**323N12.5 on GC cells by YAP1 activation was promoting immunosuppression through Treg cell differentiation and myeloid-derived suppressor cells infiltration. The biological role of RP11**–**323N12.5 in tumor growth and immunosuppression through YAP1 was also established *in vivo*** [74]**.**

### 2.7 colorectal cancer

**Colorectal cancer (CRC) is a prevalent and deadly malignancy and the third leading cause of cancer-related death worldwide. The malignancy diagnosis is commonly concluded in the late clinical stages with hopeless therapy and poor prognosis of patients. Understanding the molecular characteristics of CRC is prerequisite for discovering effective targeted therapies** [84,85]**. Hippo signaling has been documented as a key molecular pathway for homeostasis of intestinal cell proliferation through cross-talking with some non-coding RNAs, including lncRNAs** [86]**.**

#### 2.7.1 LncRNA B4GALT1-AS1

**LncRNA B4GALT1-AS1 is the antisense counterpart of B4GALT1. The biological role of lncRNA B4GALT1-AS1 in the promotion of colon cancer cell stemness has been investigated. Since B4GALT1-AS1 is one lncRNA with a high expression in colon cancer cells (**HCT116, SW480 and SW620**) compared to normal colon cells, it was chosen for further functional investigations. The knockdown of B4GALT1-AS1 in colon cancer cell lines decreased cell colony formation, but had no impact on cell viability. Moreover, B4GALT1-AS1 knockdown inhibited colon cancer cells migration, invasion, and EMT process through suppressing the expression of aldehyde dehydrogenase 1 (ALDH1) and other stemness markers. The effect of B4GALT1-AS1 on the colon cancer cells stemness was also confirmed *in vivo*. The RNA-sequencing data demonstrated that the Hippo signaling pathway was significantly upregulated in colon cancer cell lines compared to cell lines with B4GALT1-AS1 knockdown. Although B4GALT1-AS1 did not affect the YAP mRNA level, it significantly reduced the YAP protein level. They found out that YAP binds to B4GALT1-AS1, promotes YAP translocation from nuclear to cytoplasm, and decrease YAP transcriptional activity in colon cancer cells. Additionally, they showed that the inhibitory effect of B4GALT1-AS1 knockdown on cell clone formation, stemness markers expression, cell spheroid formation, and ALDH1 activity was reversed by YAP overexpression. In this regard, it was concluded that lncRNA B4GALT1-AS1 could promote colon cancer stemness via translocating YAP to nucleus to increase its transcriptional activity** [87]**.**

#### 2.7.2 LncRNA GAS5

**The YAP-binding lncRNA GAS5 was identified in CRC using RIP-sequencing. It was revealed that GAS5 expression is significantly decreased and YAPS and YTHDF3 expressions are significantly increased in 208 CRC tissues compared to normal adjacent tissues. The expression levels of the Hippo signaling-associated genes such as CTGF and CYR61, correlate with prognosis and survival of CRC patients. In CRC cell lines (**DLD1, LOVO, SW480, SW620, LS174T, HCT116, RKO and HT29**), GAS5 expression had a significant inverse correlation with YAP expression. The gain and loss of function experiments confirmed that GAS5 downregulation was associated with YAP protein nuclear localization. Briefly, GAS5 directly binds to YAP protein and promotes its translocation from nucleus to cytoplasm, enhances YAP phosphorylation, and decreases the YAP total protein via accelerating its ubiquitination and degradation** [74]**. KEGG pathway analysis confirmed the strong association of GAS5 and YAP in the Hippo pathway. The in vitro and in vivo evaluation of GAS5 impact on CRC progression demonstrated that GAS5 overexpression could suppress CRC cell proliferation, invasion, and it decreases tumor growth. YTH-domain family member 3 (YTHDF3) is a target gene of YAP that stabilizes the N6-methyladenosine (m6A) RNAs. YTHDF3 was significantly downregulated in the YAP knockdown and GAS5 overexpressed CRC cells. YAP directly binds to YTHDF3 and promotes CRC cell proliferation and invasion, while GAS5 inhibits the YAP-mediated expression of YTHDF3. Interestingly, YTHDF3 binding to m6A-modified lncRNA GAS5 enhances the degradation of GAS5** [88]**.**

#### 2.7.3 LncRNA USP2-AS1

**Recently, the biological and clinical significance of lncRNA USP2-AS1 have been investigated in colon adenocarcinoma. Findings showed that in 43 patients with CRC, the USP2-AS1 expression was significantly higher in the tumor tissues, and its level was associated with tumor grade and stage** [89]**. Previously, it was shown that two transcripts of USP2-AS1 bind to YAP1** [89]**. Li et al. confirmed that these two transcripts of USP2-AS1 bind to YAP1 in colon adenocarcinoma cell lines** SW480, SW620, and Lovo**. The *in vitro* and *in vivo* experiments revealed that USP2-AS1 overexpression enhanced colon adenocarcinoma cell proliferation, invasion, and metastasis. Moreover, USP2-AS1 overexpression decreased the *p*-LATS1, LATS1, LATS2, and p-YAP1 levels and increased total YAP1 level in colon adenocarcinoma cell lines. The Hippo pathway target genes, CTGF, CYR61, and SOX9 were inhibited by USP2-AS1 knockdown, and vice versa, which was in accordance with the effect of USP2-AS1 overexpression on inactivation of Hippo pathway** [89]**.**

#### 2.7.4 LINC00152

**LncRNA** LINC00152 **has been reported as the most downregulated lncRNA in** YAP1-suppressed CRC cell line HCT116 and the most upregulated in CRC datasets. The clinical investigations showed that 177 CRC patients with high expression of LINC00152 and FSCN1 had the worst prognosis, while if either LINC00152 or FSCN1 was downregulated; the prognosis was improved. Also, the biological function of LINC00152 has been investigated in CRC progression. LINC00152 was significantly overexpressed in CRC tissues compared to adjacent tissues in 83 patients, and its expression level was positively associated with YAP1 and CTGF levels, and poor overall survival of patients. To regulate LINC00152, YAP1 binds to TEAD1 that is predicted to have two binding sites for LINC00152 promoter. Furthermore, luciferase activity analysis demonstrated that TEAD1 binds to one of these sites and regulate the LINC00152 expression. In addition to YAP1, NF2, an upstream molecule of **t**he Hippo pathway, represses the LINC00152 expression [74]. The in vitro and in vivo analysis confirmed that LINC00152 has a role in enhancing proliferation and metastasis of CRC cells. The expression of Fascin actin-bundling protein 1 (FSCN1), that promotes CRC cell migration, was positively correlated with LINC00152 expression. Likewise, gain and loss of function experiments on LINC00152 increased and decreased the expression level of FSCN1, respectively. Both LINC00152 and FSCN have binding sites for three miRNAs, so two of them (miR-185-3p and miR-632) have more regulatory effect on FSCN1 and significantly lower expressions in the CRC tumor tissue compared to normal adjacent tissue. The luciferase activity analysis reports and in vitro experiments demonstrated that LINC00152 positively regulates FSCN1 via sponging with miR-185-3p and miR-632, and promotes cell prolifera**tion, invasion, and metastasis of CRC cells** [90]**.**

#### 2.7.5 LncRNA SNHG1

**In a recent study, Xu et al. evaluated the lncRNAs small nucleolar RNA host gene 11 (SNGH11), ZFAS1,** LINC00654, and LINC00909 circulating levels in patients with polyps, adenoma, and CRC. They revealed that the combination of these four lncRNAs provides a diagnostic tool with a satisfactory accuracy in **diagnosing CRC at early stages as well as advanced stages. The examination of molecular mechanisms of SNHG11 in CRC pathogenesis showed that SNHG11 mRNA expression was higher in CRC cell lines derived from primary tumor compared to cell lines derived from CRC metastases. The *in vitro* analysis established that SNGH11 promotes cell proliferation, migration and invasion. Moreover, knockdown of SNHG11 led to changes in EMT markers, including E-cadherin overexpression and N-cadherin downregulation in cell lines** LoVo, SW480, HCT116 and SW620**. Since EMT is related to Hippo pathway, the levels of phosphorylated-YAP and phosphorylated-LATS1 were evaluated after SNHG11 knockdown in CRC cell lines, which showed significant reductions. The *in vivo* assays also indicated that SNHG11 knockdown inhibited tumor growth in CRC mice models** [91]**.**

#### 2.7.6 LncRNA AGAP2-AS1 and LINC-PINT

**LncRNA ankyrin repeat and PH domain 2 antisense 1 (AGAP2-AS1) is one lncRNA that was previously reported to have a role in promoting tumorigenesis of gastric, lung, and breast cancers** [[92], [93], [94], [95], [96]]**. In 66 patients with CRC, lncRNA AGAP2-AS1 was significantly overexpressed in the tumor tissues compared to para-tumor normal tissues, whereas LINC-PINT expression was significantly downregulated and negatively correlated with AGAP2-AS1 expression. AGAP2-AS1 overexpression downregulated LINC-PINT expression and its inhibition upregulated LINC-PINT expression in CRC cell lines** RKO and HCT116**. Similarly, LINC-PINT overexpression and inhibition have been shown to downregulate and upregulate the AGAP2-AS1 expression, respectively. AGAP2-AS1 overexpression enhanced CRC cell proliferation, migration and invasion, whereas LINC-PINT overexpression had an inhibitory effect** [69]**.**

## 3 Future perspectives; GI cancer therapeutic strategies targeting lncRNAs

A number of functional and clinical investigations revealed the imperative role of Hippo signaling pathway in tumor development and progression. A growing number of studies have indicated biological interaction between Hippo signaling pathway and lncRNAs. Moreover, the corresponding effectors involved in the biological crosstalking regulate tumor cell functions and have clinical values; their dysregulation is correlated with clinicopathological features and might be served as promising biomarker in diagnosis and prognosis of GI cancers [74]. In this regard, Hippo signaling/lncRNA interactions also improve our insight into other various tumor properties involved in EMT and chemo‐resistance. Taken together, Hippo/lncRNAs signaling axis may be indicated as groundbreaking clinical biomarker as well as an appropriate therapeutic target for GI cancers. Since the lncRNAs structures can be straightforwardly targeted by various practicable strategies, it may be endorsed the application of these molecules as innovative drugs [74]. The advantages of these molecules as biological tools for cancer therapy include their usage in low and non-toxic doses, easy manipulation and employment, and elimination of side effects due to restore lacking. Regarding to cancer therapeutic strategies, it may modify cancer-related suppressor lncRNAs in a locus-specific mode. Also, oncogenic lncRNAs may be adjusted or suppressed for remove their epigenetic effects [74,75]. Other strategies contain interfere with biological functions, use of lncRNAs regulatory elements and modification or restoration of expression levels. Distinct most relevant nucleic acid-based approaches are antisense oligonucleotides (ASOs), locked nucleic acid GapmeRs (LNA GapmeRs), antagonist to natural antisense transcripts (antagoNATs), aptamers, small interfering RNAs (siRNAs) and short hairpin RNA (shRNAs), deoxyribozymes and ribozymes. New genome engineering tools may involve zinc finger nucleases (ZFNs), transcription activator-like effector nucleases (TALENs), and clustered regularly interspaced short palindromic repeats (CRISPR)/Cas9 system [[13], [14], [15]].

ASO-based targeting of the overexpressed lncRNAs in HCC and PC such as MALAT1, may be as an efficient approach to inhibit tumor progression, similar to what has been documented in breast [97] and lung cancer [98]. Furthermore, implication of LNAs for targeting lncRNA XIST, has probably advantages in targeting and GI anticancer therapeutic strategies [99]. AntagoNATs, as inhibitory oligonucleotides against to sense-antisense transcripts, may be implicated for reducing the epigenetic silencing effect of some oncogenic lncRNAs [100]. Thus, AntagoNATs can be designed for targeting dysregulated lncRNAs in GI cancers, such as HOTAIR and MALAT1 [101]. Mixmers and deoxy/ribozymes, another silencing approaches for targeting lncRNAs, are constructed of adjusted nucleotides to inhibit molecular interactions between lncRNAs and corresponding targets. Regarding the clinical role and significance of lncRNAs in GI cancers, such approach may be considered for targeting HOTAIR [102] and XIST [103,104]. Specific modified deoxyribozymes with a high catalytic activities have been suggested as inhibitors of N^6^-methyladenosine (m6A) modified-RNAs [105,106]. Because involving m6A modifications in lncRNAs MALAT1 [106], XIST [107], HOTAIR [108], GAS5 [88], or DANCR [109], this inhibitory structure may be as a targeting strategy for some GI cancers, including PC and CRC [110]. Sequence-specific suppression of lncRNAs MALAT1 [111], XIST [112], HOTAIR [113], NEAT1 [114] or UCA1 [115] using TALENs and CRISPR interference (CRISPRi) [116,117] may also has therapeutic potential, especially in HCC and PC.

Other approaches for targeting the common GI cancer-associated lncRNAs include aptamers, nanobodies, and RNA decoys [118]. Interfering aptamers may be used to hinder lncRNA-protein interactions [119] and inhibit HOTAIR [120] and H19 [121], in GI cancers. Nanobodies have potential to interrupt lncRNA-RNA binding protein (RBP) interaction [122]. Nanobodies can be designed to specifically target structured RNA molecules [123], and since many lncRNAs such as MALAT1 [124], NEAT1 [125], XIST [126], or HOTAIR [127] are well-identified, this approach has a potential to use in GI cancers which those lncRNAs are dysregulated. Inhibition of the target/downstream proteins by functional lncRNA-RBP complexes constructed by RNA decoys or imitators of lncRNAs may also be used for anticancer strategies [128]**.** In this regard, a mimic of HULC, one lncRNA that potentially functions in HCC cancer, can effectively regulate the function of the lncRNA and corresponding homeostasis-related signaling pathways [129].

## 4 Conclusion

Hippo signaling pathway is well-known as one of the most imperative pathways that contribute into the regulation of various cellular processes, including cell differentiation, migration and proliferation. Dysregulation of Hippo pathway resulted from aberrant expression of the corresponding molecular effectors may be involved in GI cancer development. Hence, targeting the signaling pathway may provide a potential tool for therapeutic strategy of GI cancers. However, the mechanisms of Hippo pathway regulation by non-coding RNAs in GI cancers is not yet well defined. LncRNAs, as a subclass of ncRNAs, exert numerous cellular functions and their dysregulation has confirmed to play role in carcinogenesis. The pivotal effectors of Hippo signaling, including YAP, TAZ, LATS1/2 and MST1 have been demonstrated to be targeted by lncRNAs. LncRNAs can interplay with Hippo pathway, as a key cancer-associated signaling pathway, to regulate the various cellular processes. The cross-talking between lncRNAs and Hippo signaling pathway involves in GI cancers development and progression. Considering the clinical significance of these lncRNAs, they have also been introduced as potential biomarkers in diagnostic, prognostic and therapeutic strategies in GI cancers. According to mechanisms of lncRNA-mediated regulation of Hippo signaling pathway and clinical significance, these non-coding RNAs may be served as potential targets for gene-based therapeutics in GI cancers. In order to endorse efficient therapeutic strategies for GI cancers by targeting biological axes involving lncRNAs and Hippo pathway, further functional and clinical studies in more details are required.

## Data availability statement

No data was used for the research described in the article.

## CRediT authorship contribution statement

**Farimah Fayyaz:** Writing – original draft, Formal analysis, Data curation. **Zahra Shokati Eshkiki:** Writing – review & editing, Methodology, Investigation. **Amir Reza Karamzadeh:** Investigation, Data curation. **Zahra Moradi:** Writing – original draft, Data curation. **Faezeh Kaviani:** Writing – original draft, Formal analysis, Data curation. **Abolfazl Namazi:** Project administration, Methodology, Investigation. **Roya Karimi:** Software, Methodology. **Seidamir Pasha Tabaeian:** Writing – review & editing, Validation, Methodology, Investigation, Formal analysis, Conceptualization. **Fatemeh Mansouri:** Resources. **Abolfazl Akbari:** Writing – review & editing, Writing – original draft, Visualization, Validation, Supervision, Methodology, Formal analysis, Data curation, Conceptualization.

## Declaration of competing interest

The authors declare that they have no known competing financial interests or personal relationships that could have appeared to influence the work reported in this paper.

## Abbreviations

CTGF: connective tissue growth factor
CYR61: cysteine-rich 61
GI: gastrointestinal
HNSCCs: head and neck squamous cell carcinomas
MST1/MST2: mammalian STE20-like protein kinase 1/2
LATS1/2: large tumor suppressor 1/2 (and LATS2)
YAP: Yes-associated protein
TAZ: transcriptional co-activator with PDZ-binding motif
TEAD: transcriptional enhanced associate domain
LncRNA: long non-coding RNA
ceRNA: competing endogenous RNA
ICC: intrahepatic cholangiocarcinoma
HCC: hepatocellular carcinom
MNX1-AS1: motor neuron and pancreas homeobox 1-antisense RNA1
TCGA: The Cancer Genome Atlas
GEO: Gene Expression Omnibus
PAICC: prognostic-associated ICC
LEF1-AS1: lymphoid enhancer-binding factor 1 antisense RNA 1
OSCC: oral squamous cell carcinoma
LUADT1: lung adenocarcinoma-related transcript 1
NPC: nasopharyngeal carcinoma
MALAT1: metastasis-associated lung adenocarcinoma transcript 1
PVT1: plasmacytoma variant translocation 1
UCA1: urothelial cancer associated 1
CRNDE: colorectal neoplasia differentially expressed
PRC2: polycomb repressive complex 2
GAS5: growth arrest-specific 5
EMT: epithelial-mesenchymal transition
HCG18: HLA complex group 18
FER1L4: Fer-1-like family member 4
FSCN1: Fascin actin-bundling protein 1
SNGH11: small nucleolar RNA host gene 11
**AGAP2-AS1**: **ankyrin repeat and PH domain 2 antisense 1**
MNX1-AS1: motor neuron and pancreas homeobox1 antisense RNA 1
